# Rapid, Low-Complexity, Simultaneous Bacterial Group Identification and Antimicrobial Susceptibility Testing Performed Directly on Positive Blood Culture Bottles Using Chromogenic Agar

**DOI:** 10.4269/ajtmh.22-0278

**Published:** 2022-11-14

**Authors:** F. J. Lourens Robberts, Alex Owusu-Ofori, George Oduro, Thomas K. Gyampomah, Nisha Marles, Anne T. Fox, Josh G. Chenoweth, Kevin L. Schully, Danielle V. Clark

**Affiliations:** ^1^ Independent Consultant, Stellenbosch, South Africa;; ^2^ Department of Clinical Microbiology, School of Medical Sciences, Kwame Nkrumah University of Science and Technology, Kumasi, Ghana;; ^3^ Komfo Anokye Teaching Hospital, Kumasi, Ghana;; ^4^ American Society for Microbiology, Global Public Health Programs, Washington, District of Columbia;; ^5^ Naval Medical Research Unit-3 Ghana Detachment, Accra, Ghana;; ^6^ The Austere Environments Consortium for Enhanced Sepsis Outcomes, The Henry M. Jackson Foundation for the Advancement of Military Medicine Inc., Bethesda, Maryland;; ^7^ The Austere Environments Consortium for Enhanced Sepsis Outcomes, Naval Medical Research Centre, Fort Detrick, Maryland

## Abstract

The use of positive blood culture bottles for direct disk diffusion susceptibility testing (dDD), together with chromogenic culture limited to groups of pathogens for antimicrobial susceptibility testing interpretation may provide a means for laboratories-in-development to introduce rapid abbreviated blood culture testing. We assessed the performance of dDD on Chromatic MH agar using contrived positive blood culture bottles and compared findings with current standard practice. Furthermore, we characterized the growth of 24 bacterial and 3 yeast species on Chromatic MH agar with the aid of rapid spot tests for same-day identification. The coefficient of variation for reproducibility of dDD of four reference strains in 4 to 10 replicates (238 data points) ranged from 0% to 16.3%. Together with an additional 10 challenge isolates, the overall categorical agreement was 91.7% (351 data points). The following bacteria were readily identifiable: cream/white *Staphylococcus aureus*, coagulase-negative staphylococci, *Streptococcus pyogenes*; turquoise *Streptococcus agalactiae*, enterococci, *Listeria monocytogenes*; mauve *Escherichia coli, Shigella sonnei, Citrobacter freundii*; dark-blue *Klebsiella* and *Enterobacter*; green *Pseudomonas aeruginosa*; and brown *Proteus*. Clear colonies were seen with *Salmonella*, *Acinetobacter*, *Burkholderia*, and *Yersinia enterocolitica* (turns pink). Our study suggests that Chromatic MH for dDD may show promise as a rapid, clinically useful presumptive method for overnight simultaneous identification and antimicrobial susceptibility testing. However, there is a need to optimize the medium formulation to allow the recovery of *Streptococcus pneumoniae* and *Haemophilus influenzae*.

## INTRODUCTION

Access to reliable diagnostic microbiologic testing is limited in austere environments, leading to potential misdiagnosis, inadequate treatment, increased mortality, and the inability to determine the prevalence and dynamics of disease etiology.^1^ A systematic review of community-acquired bloodstream infections in sub-Saharan Africa yielded mortality rates of 19% and 39% for sepsis and severe sepsis, respectively.^2^ Sepsis can lead to lifelong disability in many survivors, and the cost and complexity required for effective microbiologic testing can be a deterrent to implementation.^3^^–^^6^ Exacerbating these challenges in austere environments is the increasing degree of analytical specificity demanded by ever-increasing bacterial taxonomic refinements and technological sophistication.^7^ Laboratory analyses fall across a spectrum, from rapid testing that provides presumptive evidence for clinical decision making, to advanced analytical reference testing for confirmation and the advancement of medical sciences. There is a growing inequality between the emerging literature in clinical bacteriology, generated with technologically advanced instruments, and what can be implemented sustainably in austere environments. More research is needed to enable laboratories-in-development to provide quality microbiologic testing that has clinical utility and supports public health.

Most positive blood culture bottles require 1 to 3 days of incubation before microbial growth is detected using manual or automated continuous-monitoring instruments.^8^^,^^9^ Thereafter, current standard laboratory practice requires the recovery and preparation of a pure bacterial isolate at a standardized inoculum density for subsequent species-level identification and antimicrobial susceptibility testing (AST)— adding an additional 1 to 2 days of analysis. Such delays in turnaround time, together with the direct costs associated with blood cultures and analytical methods, create a barrier to implementation of blood culture testing at laboratories-in-development (personal observations).

Advanced technologies for identification and AST directly from positive blood culture bottles include matrix-assisted laser desorption/ionization time-of-flight mass spectrometry, nucleic acid amplification tests, microfluidics, microcalorimetry, and next-generation sequencing.^10^ However, in addition to interpretive and analytical challenges, cost and infrastructure requirements prevent sustainable implementation. Rapid, potentially more sustainable, phenotypic culture-based approaches remain an attractive alternative. In the late 1970s and early 1980s, promising results were reported with the direct use of positive blood culture bottle broth for disk diffusion AST (direct disk diffusion [dDD]) as compared with standard disk diffusion (DD) using isolated colonies.^11^^–^^15^ More recently, dDD was found to compare favorably with newly introduced automated microbroth dilution instruments.^16^ Although concern about regulatory restrictions may have hampered widespread use of dDD in the absence of consensus guidelines, more recent studies confirming accuracy and reproducibility call for a reinvigoration of dDD studies.^10^^,^^17^^–^^19^

DD zone size interpretations of the Clinical Laboratory Standards Institute (CLSI) and the European Society Clinical Microbiology and Infectious Diseases identifies only seven groups of non-fastidious organisms for zone size interpretation: the Enterobacterales, *Pseudomonas aeruginosa*, *Staphylococcus*, *Enterococcus*, *Acinetobacter*, the *Burkholderia cepacia* complex, and *Stenotrophomonas maltophilia*. Within these groups, differentiating the coagulase-negative staphylococci from *Staphylococcus aureus*, and *Salmonella* and *Shigella* within the Enterobacterales is currently required for result interpretation.^20^^,^^21^ Globally and in sub-Saharan Africa, the most common bacterial causes of sepsis are *Escherichia coli*, *Klebsiella*, *Salmonella*, *P. aeruginosa*, *Acinetobacter*, *S. aureus*, beta-hemolytic *Streptococcus*, and *Streptococcus pneumoniae*.^2^^,^^22^^,^^23^ Limiting the identification of isolates to CLSI or European Society Clinical Microbiology and Infectious Diseases interpretive groups provides an opportunity for laboratories-in-development to introduce rapid abbreviated blood culture testing with meaningful clinical utility.

Chromogenic agar is an accurate, rapid, and cost-effective alternative media to traditional bacteriological culture media for isolation and differentiation of urinary tract pathogens *E. coli*, *Klebsiella*–*Enterobacter–Serratia* group, the *Proteus–Providencia*–*Morganella* group, *P. aeruginosa*, *Acinetobacter*, *Staphylococcus*, *Enterococcus*, and beta-hemolytic *Streptococcus*.^24^^–^^29^ In addition, Singh and Bhunia^30^ used optical laser technology for label-free, real-time, on-plate colony screening to differentiate clinically relevant pathogens accurately without the need for additional tests. The chromogens incorporated into chromogenic bacteriological media are typically analogs of naturally occurring di- and oligosaccharides, peptides, or esters. Such chromogens are comprised of two parts: the first is recognized by a transport system and a hydrolytic enzyme, often capable of operon induction; and a second molecule, the chromophore, when hydrolyzed leads to dimerization, chelation of metal ions, or electronic rearrangement, resulting in a shift in light absorption seen as a color change.^31^^,^^32^ Chromatic MH agar (Liofilchem, Roseto degli Abruzzi, Italy) is a non-selective, Mueller–Hinton-based agar incorporating a proprietary blend of chromogenic substrates that, when cleaved by microbial enzymes, result in color changes that correlate to clinically relevant pathogens. Chromatic MH agar has been used as substitute for traditional Mueller–Hinton agar for DD and gradient strip AST and performs well for the simultaneous isolation and direct AST of non-fastidious pathogens in urine,^33^ respiratory specimens,^34^ and vascular prosthetic graft specimens.^35^ Combined, these characteristics raise the possibility of using Chromatic MH as a solid agar medium for isolation, identification, and dDD of positive blood culture bottle broth with reduced turnaround time and a minimum of reagents, media, labor, and complexity, rendering this an attractive option in resource-limited settings.

The Austere Environments Consortium for Enhanced Sepsis Outcomes is engaged in observational studies of sepsis at Komfo Anokye Teaching Hospital (KATH), Kumasi, Ghana, where our collaboration is seeking to enable sustainable laboratory approaches that may be implemented in resource-constrained environments. To this end, we assessed the performance of Chromatic MH agar for dDD from contrived positive blood culture bottles and compared findings with current standard practice using subcultured isolates and the CLSI method. Furthermore, we characterized the growth of 24 different bacterial and 3 yeast species on Chromatic MH agar with the aid of rapid spot tests for same-day identification.

## MATERIALS AND METHODS

### Chromogenic culture characterization.

Twenty-four bacterial and three yeast reference strains were obtained from the American Type Culture Collection (ATCC) and are detailed in Table 1. Lyophilized strains were revived and sub-cultured serially twice before inoculation onto Chromatic MH agar plates. Plates were incubated at 37°C in room air or a candle jar (*S. pneumoniae* and *Haemophilus influenzae*) and evaluated for chromogenic reactions and colonial growth characteristics daily for up to 72 hours. For *Erysipelothrix rhusiopathiae, Yersinia enterocolitica, and Pseudomonas fluorescens*, plates were transferred from 37°C to room-temperature incubation after the initial 24 hours. Photos of agar plates were taken using a mobile phone. Rapid identification reagents were used on colonies taken directly from Chromatic MH to aid differentiation: catalase (3% hydrogen peroxide; LabChem Inc., Zelienople, PA), slide coagulase (CoaguStaph; Hardy Diagnostics, Santa Maria, CA); and the following in-house prepared reagents (Sigma-Aldrich, St. Louis, MO): rapid spot indole filter paper method using 1% 4-dimethylaminocinnamaldehyde, spot oxidase filter paper using Kovác’s 1% tetramethyl-p-phenylenediamine dihydrochloride solution, 0.02% L-pyrrolidonyl-β-naphthylamide visualized with 4-dimethylaminocinnamaldehyde, spot filter paper with 0.02% esculin visualized with 1% ferric ammonium citrate, and sulfur–indole–motility medium (Criterion; Hardy Diagnostics, Santa Maria, CA).^36^^,^^37^ Testing and quality control (QC) were performed according to York et al.^38^

**Table 1:** Strains and isolates

Number	Strains and isolates
1	*Enterococcus faecalis* ATCC 29212
2	*Staphylococcus aureus* subsp. *aureus* ATCC 25923
3	*Staphylococcus epidermidis* ATCC 12228
4	*Streptococcus agalactiae* ATCC 12386
5	*Streptococcus pneumoniae ATCC 6305*
6	*Streptococcus pyogenes* ATCC 19615
7	*Erysipelothrix rhusiopathiae* ATCC 19414
8	*Listeria monocytogenes* ATCC 19115
9	*Citrobacter freundii* ATCC 8090
10	*Cronobacter sakazakii* ATCC 29544
11	*Escherichia coli* ATCC 35218
12	*E. coli* ATCC 25922
13	*Haemophilus influenzae* ATCC 10211
14	*Klebsiella pneumoniae* subsp. *pneumoniae* ATCC 13883
15	*Proteus mirabilis* ATCC 12453
16	*Salmonella enterica* Enteritidis ATCC 13076
17	*Salmonella enterica* Typhimurium ATCC 14028
18	*Serratia marcescens* ATCC 43861
19	*Yersinia enterocolitica* subsp. *enterocolitica* ATCC 9610
20	*Acinetobacter baumannii* ATCC 19606
21	*Burkholderia cepacia* ATCC 25416
22	*Pseudomonas aeruginosa* ATCC 27853
23	*Pseudomonas fluorescens* ATCC 13525
24	*Stenotrophomonas maltophilia* ATCC 13637
25	*Cryptococcus neoformans* ATCC 32045
26	*Candida krusei* ATCC 34135
27	*Candida albicans* ATCC 10231
Challenge isolates*	
1	*S. aureus*
2	*Klebsiella aerogenes*
3, 4, 5	*K. pneumoniae* (3 isolates)
6	*Salmonella enteritidis*
7	*A. baumannii*
8, 9	*P. aeruginosa* (2 isolates)
10	*Vibrio parahaemolyticus*

ATCC = American Type Culture Collection.

* Challenge isolates consisted of clinical isolates from Komfo Anokye Teaching Hospital and isolates from external proficiency surveys.

### Standard AST.

We performed DD QC testing on Chromatic MH, Mueller–Hinton Agar (Thermo Scientific Oxoid; Thermo Fisher Scientific, Waltham, MA), and antimicrobial disks as described by CLSI using *S. aureus* ATCC 25923, *E. coli* ATCC 25922, *E. coli* ATCC 35218, and *P. aeruginosa* ATCC 27853.^20^ In addition, we subjected 10 challenge isolates (Table 1) to DD on both agar media.

### Contrived blood culture-positive sample preparation.

Blood culture bottles (Bactec Plus Aerobic/F 30 mL; BD Diagnostics, Sparks, MD) were inoculated with approximately 15 colony forming units (CFU) of each of the 4 QC strains and the 10 challenge isolates. Briefly, a bacterial suspension approximating a 0.5 McFarland standard (1.5 × 10^8^ CFU/mL) was prepared in normal saline from an overnight solid agar culture and diluted 1:100 by transferring 100 μL into 9.9 mL trypticase soy broth (BD Bacto; Becton, Dickinson and Company, Franklin Lakes, NJ). A further 2 × 1:100 serial dilution in trypticase soy broth was made to yield a final concentration of approximately 1.5 × 10^2^ CFU/mL. From this, 0.1 mL was inoculated (approximately 15 CFU) with a tuberculin syringe into a blood culture bottle. A total of 8 mL of expired human blood collected in citrate–phosphate–dextrose–adenine obtained from the KATH blood bank was added to each bottle and incubated in either a Bactec 9240 and/or FX40 instrument (BD Diagnostics, Sparks, MD). Replicates were prepared on different days using two lots of blood culture bottles: 4 replicates of *S. aureus* ATCC 25923, 8 replicates of each of *E. coli* ATCC 25922 and *E. coli* ATCC 35218, and 10 replicates of *P. aeruginosa* ATCC 27853. A single preparation was made of each of the 10 challenge isolates. Blood bottles were removed and processed for Gram stain and culture within 6 hours of the instrument positive signal.

### Direct disk diffusion susceptibility testing.

Antimicrobial susceptibility disks used (Table 2) were obtained from two manufacturers (BBL Sensi-Disc, BD Diagnostics, Sparks, MD; and Thermo Scientific Oxoid, Thermo Fisher Scientific, Waltham, MA). Briefly, 4 mL of broth was transferred from a positive blood culture bottle into a sterile Bijou bottle. From the Bijou bottle, Chromatic MH agar plates were inoculated using a sterile cotton-tipped swab rubbed in three directions while rotating the swab before antimicrobial disks were placed. Antimicrobial susceptibility agar plates were incubated in ambient air at 37°C for 18 to 20 hours before inhibition zone size measurements were taken with a clear ruler, against a black background with reflected light, from the top of the agar plate with lids removed. For the QC strains, the zone size diameters were compared with the acceptable QC ranges established for each specific antimicrobial disk and QC strain combination.^20^ Zone size measurements obtained from dDD onto Chromatic MH agar were compared with those obtained from the standard CLSI DD method using Mueller–Hinton agar.^20^

**Table 2:** Antimicrobial susceptibility disk

Antimicrobial agent and disk content	Abbreviation
Amoxicillin–clavulanate 20/10 μg	AMC
Ampicillin 10 μg	AMP
Azithromycin 15 μg	AZM
Cefazolin 30 μg	CZ
Cefoxitin 30 μg	FOX
Cefpodoxime 10 μg	CPD
Ceftazidime 30 μg	CAZ
Ceftriaxone 30 μg	CRO
Cefuroxime 30 μg	CXM
Chloramphenicol 30 μg	C
Ciprofloxacin 5 μg	CIP
Clindamycin 2 μg	CC
Erythromycin 15 μg	E
Gentamicin 10 μg	GM
Imipenem 10 μg	IMP
Meropenem 10 μg	MEM
Penicillin 10 U	P
Rifampicin 5 μg	RA
Tetracycline 30 μg	TE
Trimethoprim–sulfamethoxazole 1.25/23.75 μg	SXT
Vancomycin 30 μg	VA
Nitrofurantoin 300 μg	NIT

### Statistical analysis.

The reproducibility of dDD on Chromatic MH agar was assessed by comparing the mean, SD, coefficient of variation (CV), range, and 95% CI (Student’s *t*-test for small sample size, rounded to the nearest whole millimeter) of zone sizes obtained. The precision (reproducibility) categorical agreement was calculated as the number of categorical result matches/total number of results for all organisms and drugs combined.^39^ The categorical agreement (CA), very major error (VME) rate, major error rate, and minor error rate for dDD was determined by comparison to standard DD for the 10 challenge isolates and the 4 QC strains combined against all drugs tested. VME (false-susceptible) rates were calculated as number of results resistant by DD but susceptible by dDD/total number of resistant results. Major error (false-resistant) rates were calculated as number susceptible by DD but resistant by dDD/number of susceptible isolates. Minor errors were defined as one result yielding an intermediate category, and the other either a susceptible or resistant result.^39^

## RESULTS

### Chromogenic culture characterization.

To assess the utility of Chromatic MH agar for the isolation and differential identification of pathogens commonly encountered in blood cultures, we evaluated 24 bacteria and 3 yeasts (Table 3). Plates with insufficient growth for characterization were reincubated and inspected daily for an additional 48 hours. No growth was obtained for *S. pneumoniae* and *H. influenzae*. All other organisms yielded sufficient growth on Chromatic MH agar after overnight incubation for characterization except for *E. rhusiopathiae*, *Y. enterocolitica*, *P. fluorescens*, and *Cryptococcus neoformans*, which required 72 hour for faint, clear growth of tiny colonies. Photos of chromogenic cultures are shown in Supplemental Figures S1 through S24. Rapid tests were used to differentiate further between species with the same color when possible (Table 3).

**Table 3:** Chromogenic culture and differentiation

Culture result	Rapid test	Organism
Gram positive		
Cream/white	Cat+, Coag+	*Staphylococcus aureus*
	Cat+, Coag–	*Staphylococcus epidermidis*
	Cat–	*Streptococcus pyogenes*
Turquoise	Cat–, Esc+, PYR+	*Enterococcus faecalis*
	Cat–, Esc–, PYR–	*Streptococcus agalactiae*
	Cat+, Esc+, PYR–	*Listeria monocytogenes*
Clear faint	S+, Ind–, M+	*Erysipelothrix rhusiopathiae*
Gram negative		
Mauve	PYR–, Ind+	*Escherichia coli*
	PYR–, Ind–	*Shigella sonnei*
	PYR+, Ind–	*Citrobacter freundii*
Dark blue	–	*Klebsiella pneumoniae*
	–	*Cronobacter sakazakii*
	–	*Klebsiella aerogenes*
Turquoise	Ox–, PYR+	*Serratia marcescens*
	Ox–, PYR–	*Stenotrophomonas maltophilia*
	Ox+, PYR–	*Vibrio parahaemolyticus*
Green	–	*Pseudomonas aeruginosa*
Clear	Ox+, PYR–	*Burkholderia cepacian*
	Ox–, PYR–	*Acinetobacter baumannii*
	Ox–, PYR–	*Salmonella enterica*
	Ox–, PYR+, purple at RT	*Yersinia enterocolitica*
Clear	Faint at 72h at RT	*Pseudomonas fluorescens*
Brown precipitate	–	*Proteus mirabilis*
Yeast		
Cream/white	–	*Candida albicans*
	–	*Candida krusei*
Clear, faint at 72 hours	–	*Cryptococcus neoformans*

Cat = catalase; Coag = coagulase; Esc = esculin; Ind = indole; M = motility; Ox = Oxidase; PYR = pyrrolidonyl arylamidase; RT = room temperature; S = sulfur.

### Standard AST.

Prior to examining the potential of dDD on Chromatic MH agar, we first performed QC testing of type strains using standard DD methods described elsewhere.^20^ QC of standard DD of antimicrobial disks using Mueller–Hinton agar yielded seven instances of zone sizes outside of the acceptable range: *S. aureus* ATCC 25923 against cefoxitin 30 μg (FOX; +1 mm), meropenem 10 μg (MEM; +1 mm), and tetracycline 30 μg (+4 mm); *E. coli* ATCC 25922 against chloramphenicol (C; +1 mm), rifampicin 5 μg (–1 mm); and *P. aeruginosa* ATCC 27853 against gentamicin 10 μg (GM; +3 mm) and MEM (+1 mm). Upon retesting, all QC failures were resolved. In contrast, the same strains and disks yielded no zone sizes out of range with Chromatic MH (data not shown).

### Direct disk diffusion susceptibility testing.

Having established that standard DD performs favorably well on Chromatic MH agar, we next sought to compare categorical agreement between dDD from spiked positive blood culture bottles with standard DD. For the 10 challenge isolates (Supplemental Table S1), the categorical agreement with 113 data points was 79.6%. No VMEs were seen. Three major errors (3 of 61, 4.9%) were seen: one for each of two *K. pneumoniae* isolates imipenem 10 μg (IMP) was found resistant by dDD on Chromatic MH (both 19 mm) versus susceptible by DD on Mueller-Hinton agar (MHA) (29 and 30 mm); and for the third *K. pneumoniae* isolate, FOX was resistant (14 mm) versus susceptible (21 mm). The minor error rate was 17.7% (20 of 113).

To assess reproducibility of dDD on Chromatic MH agar we prepared 4 to 10 replicates of the 4 QC strains in contrived blood culture bottles and subjected them to testing: *S. aureus* ATCC 25923 tested against 21 antimicrobial agents in 4 replicates yielded 4 of 84 zone sizes outside acceptable QC ranges (Supplemental Table S2): one instance of vancomycin 30 μg; *E. coli* ATCC 25922 tested against 17 agents in 8 replicates yielded zone sizes outside the acceptable QC range for cefazolin 30 μg (CZ; v5 replicates), ciprofloxacin 5 μg (4 replicates), amoxicillin–clavulanate 20/10 μg (AMC; 3 replicates), MEM (3 replicates), trimethoprim–sulfamethoxazole 1.25/23.75 μg (3 replicates), nitrofurantoin 300 μg (3 replicates), ceftriaxone 30 μg (2 replicates), cefpodoxime 10 μg (1 replicate), cefuroxime 30 μg (1 replicate), C (1 replicate), GM (1 replicate), IMP (1 replicate), rifampicin 5 μg (1 replicate), and tetracycline 30 μg (1 replicate); *E. coli* ATCC 35218 tested against 2 agents in 8 replicates yielded zone sizes outside the QC range for AMC (2 replicates); *P. aeruginosa* tested against 6 agents in 10 replicates yielded zone sizes outside the QC range for GM (2 replicates) and ceftriaxone 30 μg (1 replicate). The imprecision of replicate zone sizes measured for 238 data points ranged from 0% to 16.3% CV. CA interpretations showed no VMEs. However, six instances of minor errors were observed for *E. coli* ATCC 259223: three of eight AMC replicates yielded intermediate results (expected susceptible), one of eight CZ yielded resistant (expected susceptible or intermediate), and two of eight C yielded intermediate results (expected susceptible). The precision (reproducibility) CA yielded 97.5% (232 of 238) categorical result matches. During analysis for CA calculations, it was noted that the acceptable CLSI zone sizes in particular instances encompassed two categories: for *S. aureus* ATCC 25923 the acceptable erythromycin zone size range is 22 to 30 mm, which encompass the breakpoints susceptible ≥ 23 mm, and intermediate 14 to 22 mm. Similarly, for *E. coli* ATCC 25922, the AMC and CZ acceptable QC ranges yield susceptible or intermediate categories, and for *E. coli* ATCC 35218 against AMC, either susceptible or intermediate is acceptable.^20^ This raises a question about the appropriateness of the type strains recommended for QC, and the meaning of measurement variations as currently defined.

## DISCUSSION

Of the 24 bacteria cultured onto Chromatic MH agar, 19 yielded sufficient growth for identification after overnight incubation. *Erysipelothrix rhusiopathiae*, *P. fluorescens*, and *Y. enterocolitica* required re-incubation for an additional 24 to 48 hours at room temperature for sufficient growth, consistent with culture on routinely used non-selective clinical bacteriological media. As expected, *S. pneumoniae* and *H. influenzae* failed to grow, because no blood, hemoglobin, or X and V factors were added to the medium. With simple rapid spot identification reagents, organisms were readily assigned to the appropriate CLSI interpretive categories. *Candida* was readily recovered on Chromatic MH agar and can be differentiated with the germ tube test. *Cryptococcus* growth was scant.

QC testing of media and disks showed that Chromatic MH agar used with the standard CLSI DD method yielded no QC failures, whereas traditional MHA yielded seven instances requiring retesting. This may be a result of enhanced readability of zone size edges with a chromogenic color reaction as opposed to traditional MHA. Ease of zone size readability is beneficial for initial skills development.

The performance of dDD onto Chromatic MH agar was satisfactory in our study. The overall CA between standard dDD and DD was 91.7%, which satisfies the Cumitech-recommended 90%.^39^ However, we did observe a lower CA (79.6%) in the subset of 10 challenge isolates. Of note, three major errors were seen with *K. pneumoniae* affecting IMP and FOX results, possibly a result of an inoculum effect. The precision (CV, 0–16%) and reproducibility CA (97.5%) of replicate measures zone size measures were high.

There have been at least eight larger previous studies evaluating dDD on positive blood culture bottles since 1976, comprising a total of 3,949 blood culture isolates and 28,126 organism–antibiotic combinations. From these studies, CA ranged from 93.9% to 97.7%, of which VMEs comprised 0% to 1.6%—and major errors, 0.03% to 2.1%—supporting the direct use of positive blood culture broth for disk diffusion susceptibility testing.^10^^,^^11^^,^^13^^,^^14^^,^^16^^–^^19^ Our study suggests that the use of Mueller–Hinton-based chromogenic media for dDD of positive blood culture bottles shows potential for rapid, clinically useful presumptive overnight simultaneous identification and AST, but requires further evaluation with routine blood culture samples from patients. There is, however, a need to optimize the medium formulation to allow the recovery of *S. pneumoniae* and *H. influenzae*, and to assess the potential and limitations for identifying *Neisseria meningitidis* and/or ruling out select agents of concern for laboratory exposure.

## Supplemental files


Supplemental materials
