# Supplementary material for: Rapid, Low-Complexity, Simultaneous Bacterial Group Identification and Antimicrobial Susceptibility Testing Performed Directly on Positive Blood Culture Bottles Using Chromogenic Agar
Source: Am J Trop Med Hyg. 2022 Nov 14;107(6):1302–7. doi: 10.4269/ajtmh.22-0278 (PMC9768277; doi:10.4269/ajtmh.22-0278)
Supplement: Supplementary file 1 [file tpmd220278.SD1.pdf]

Supplementary Figures

Figure 1. *Staphylococcus aureus* subsp. *aureus* ATCC 25923

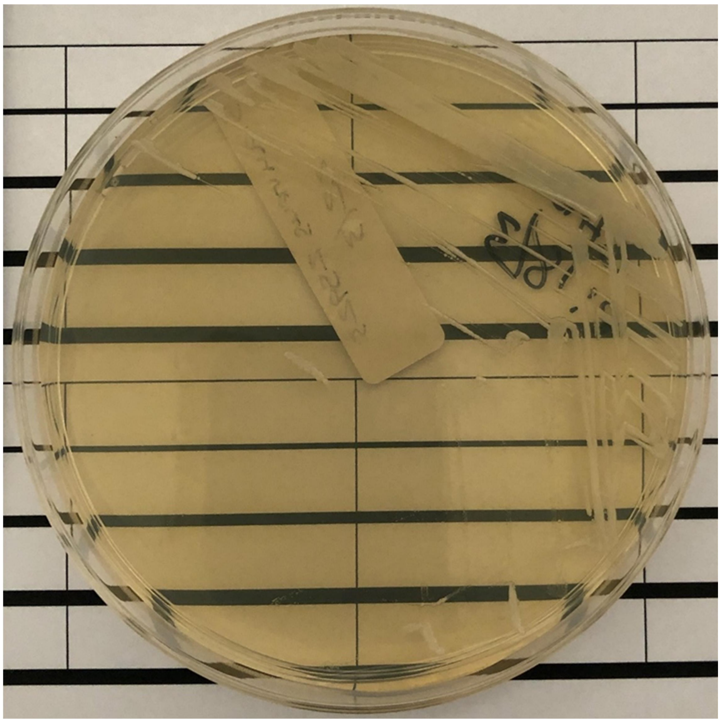

Figure 2. *Streptococcus pyogenes* ATCC 19615

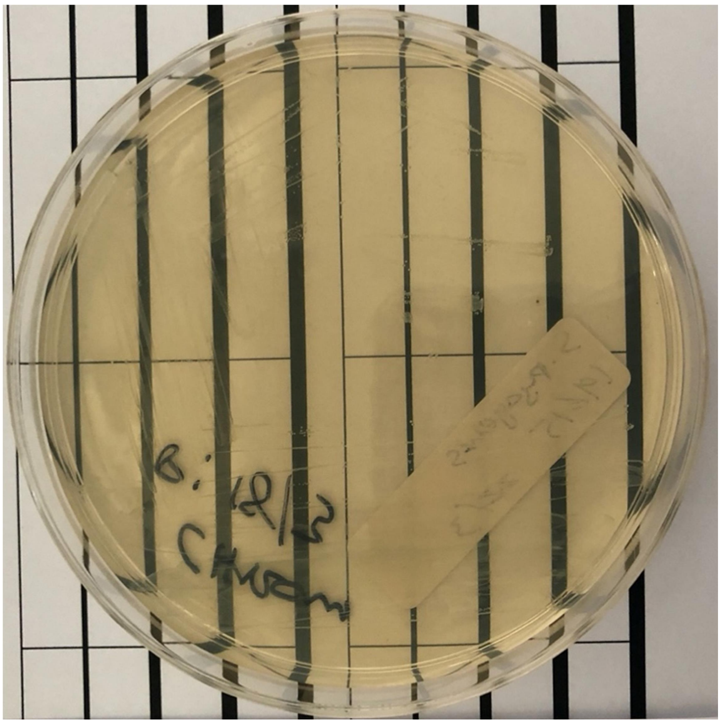

Figure 3. *Erysipelothrix rhusiopathiae* ATCC 19414

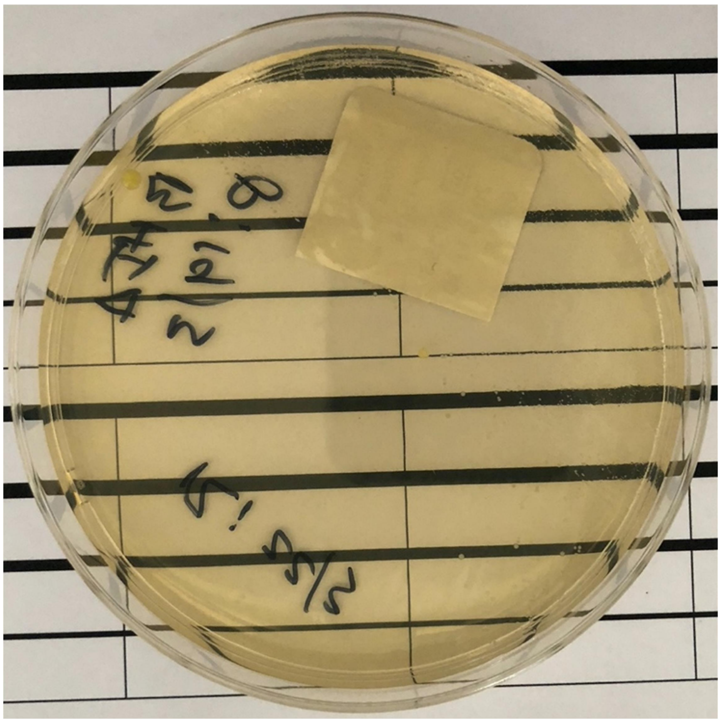

Figure 4. *Enterococcus faecalis* ATCC 29212

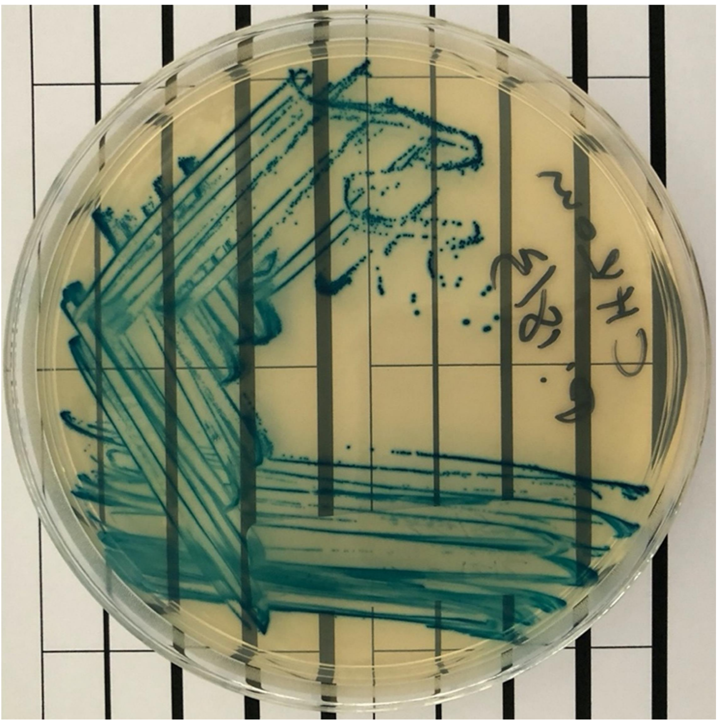

Figure 5. *Streptococcus agalactiae* ATCC 12386

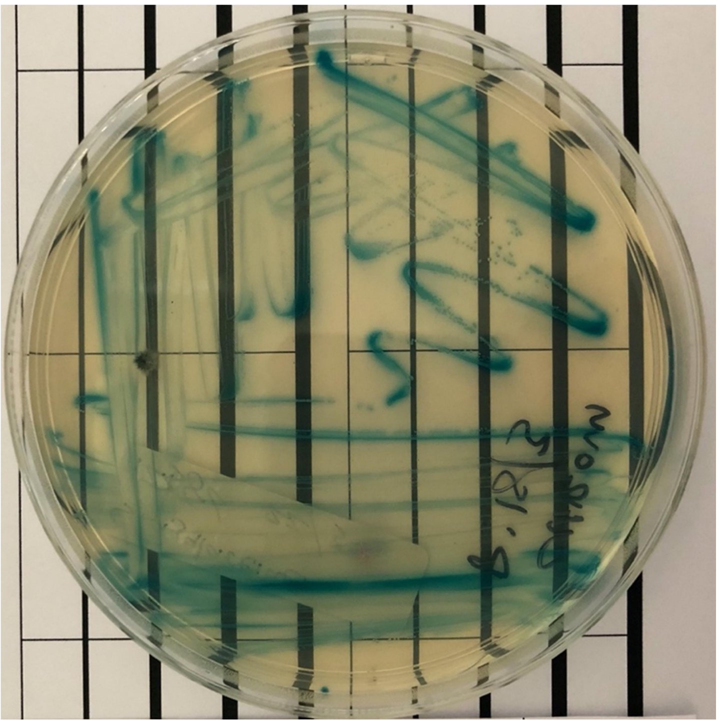

Figure 6. *Listeria monocytogenes* ATCC 19115

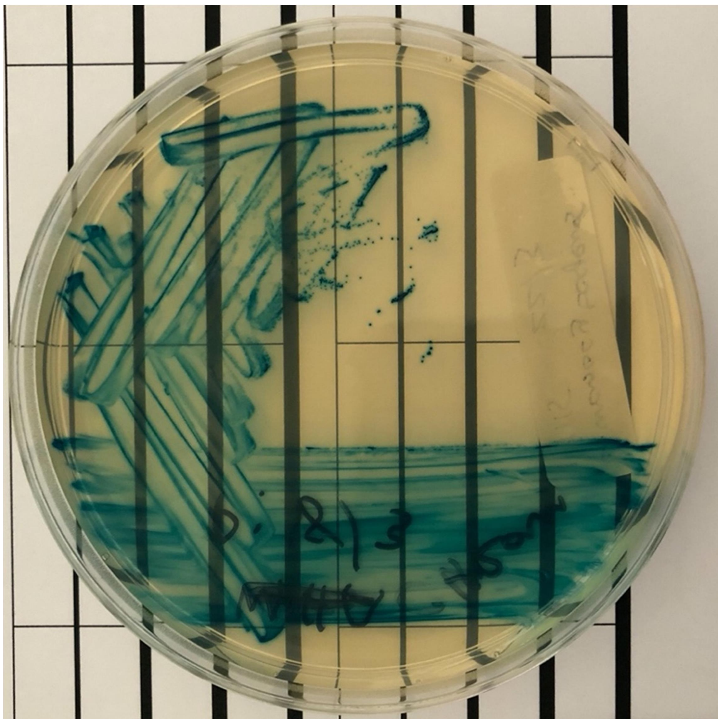

Figure 7. *Escherichia coli* ATCC 35218

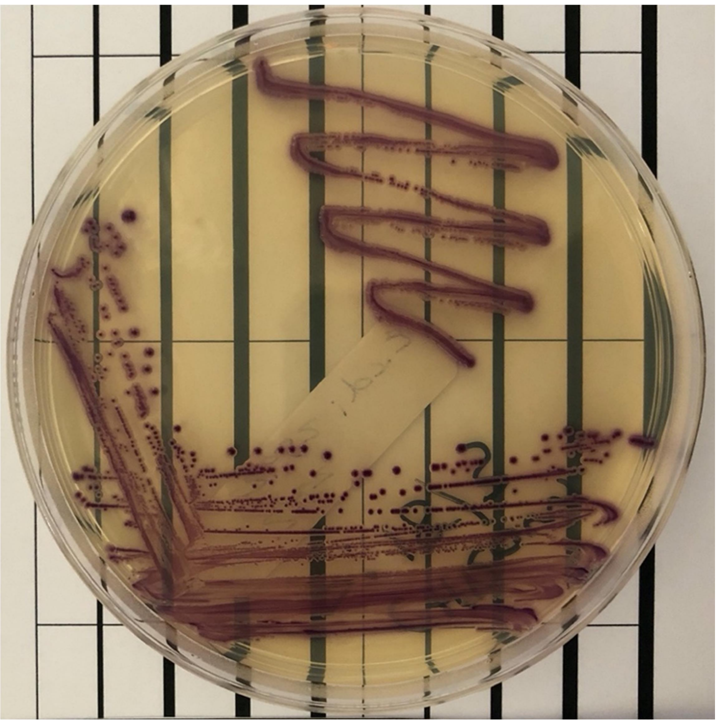

Figure 8. *Citrobacter freundii* ATCC 8090

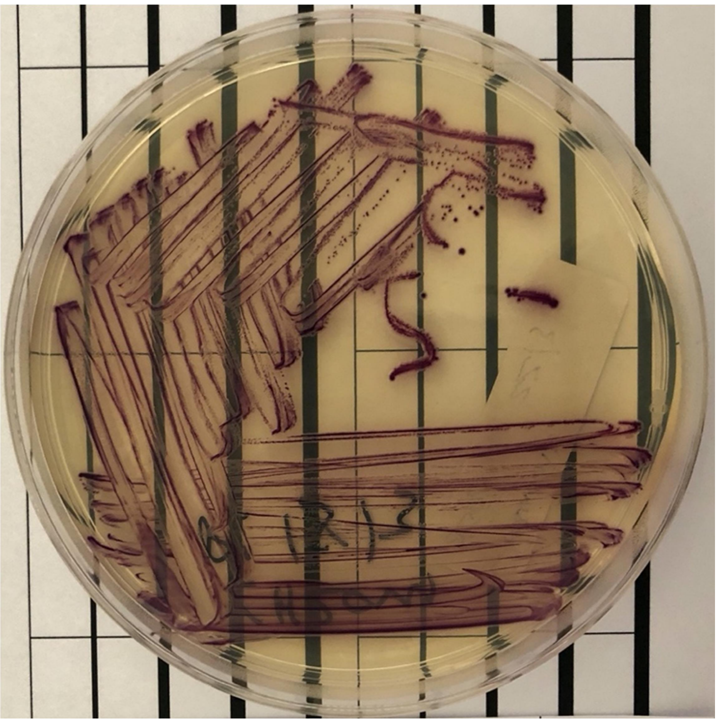

Figure 9. *Klebsiella pneumoniae* subsp. *pneumoniae* ATCC 13883

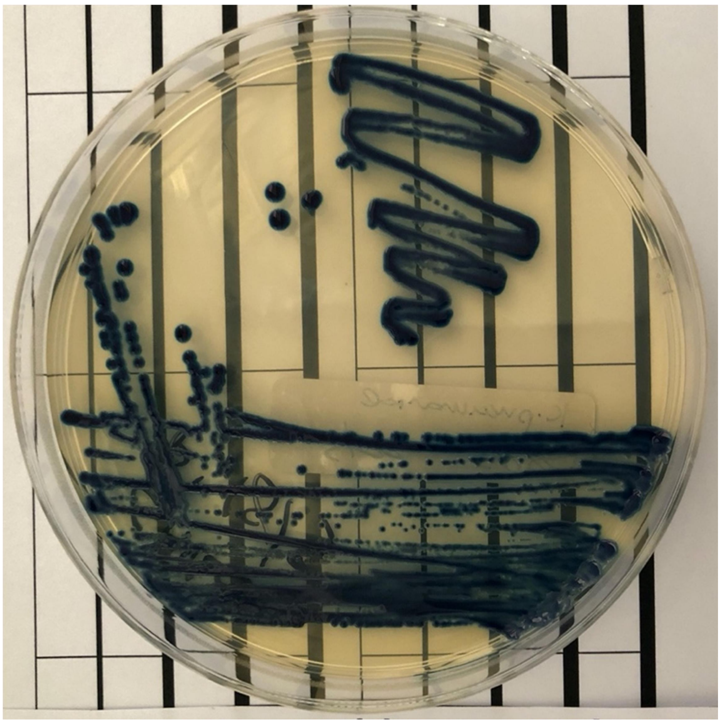

Figure 10. *Cronobacter sakazakii* ATCC 29544

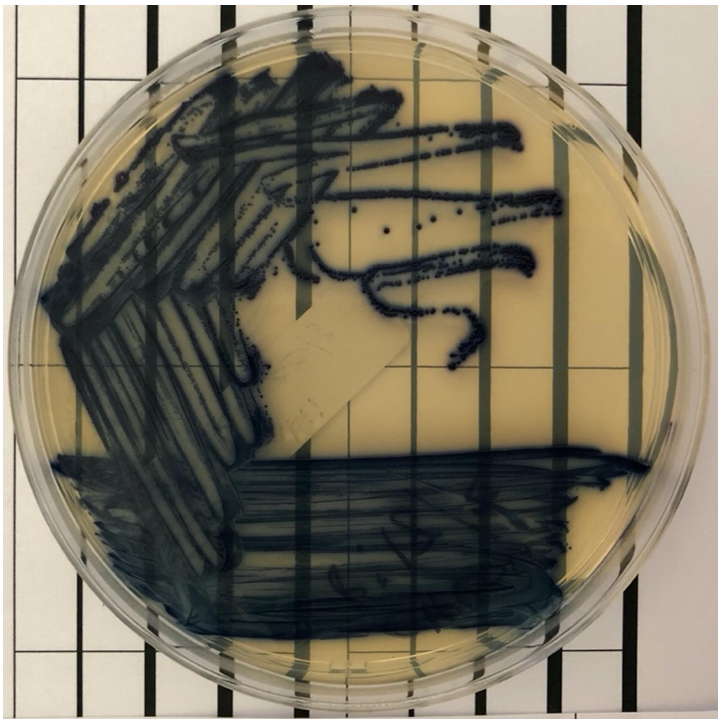

Figure 11. *Serratia marcescens* ATCC 43861

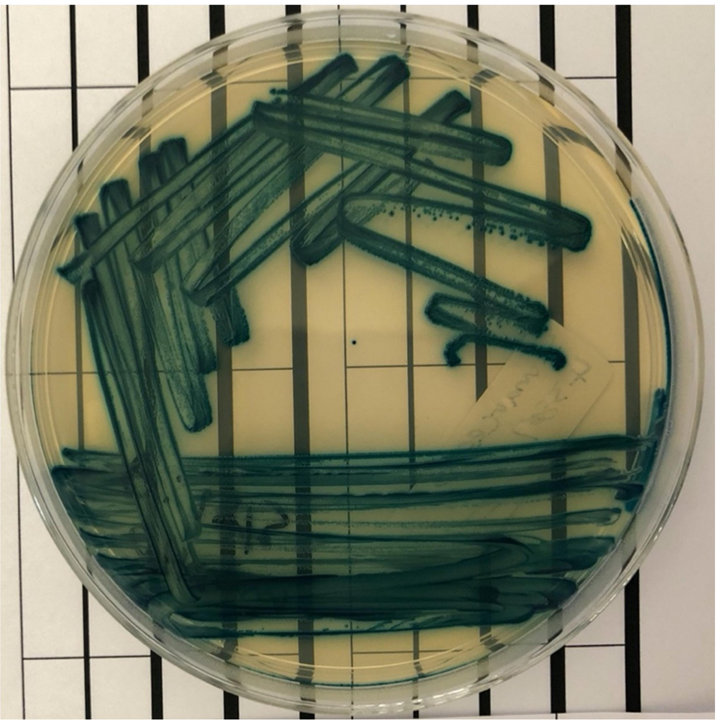

Figure 12. *Stenotrophomonas maltophilia* ATCC 13637

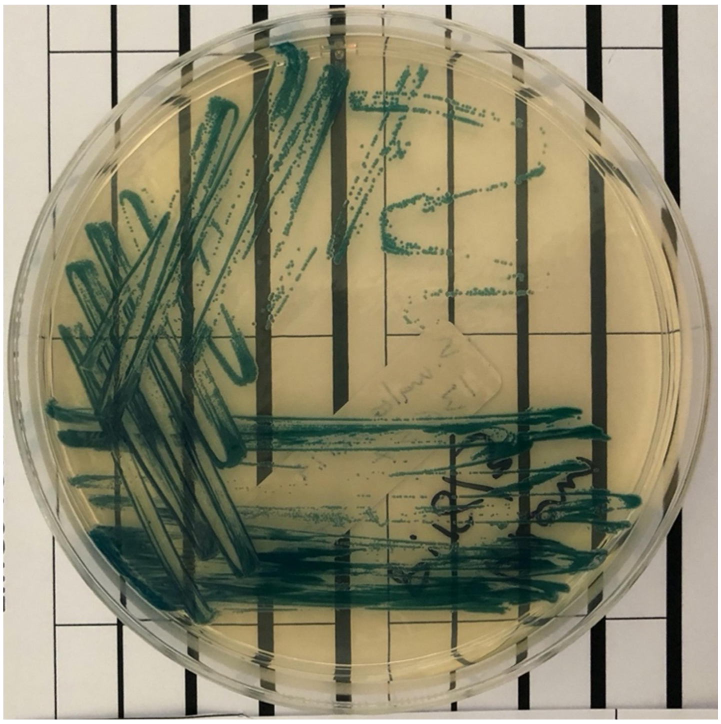

Figure 13. *Pseudomonas aeruginosa* ATCC 27853

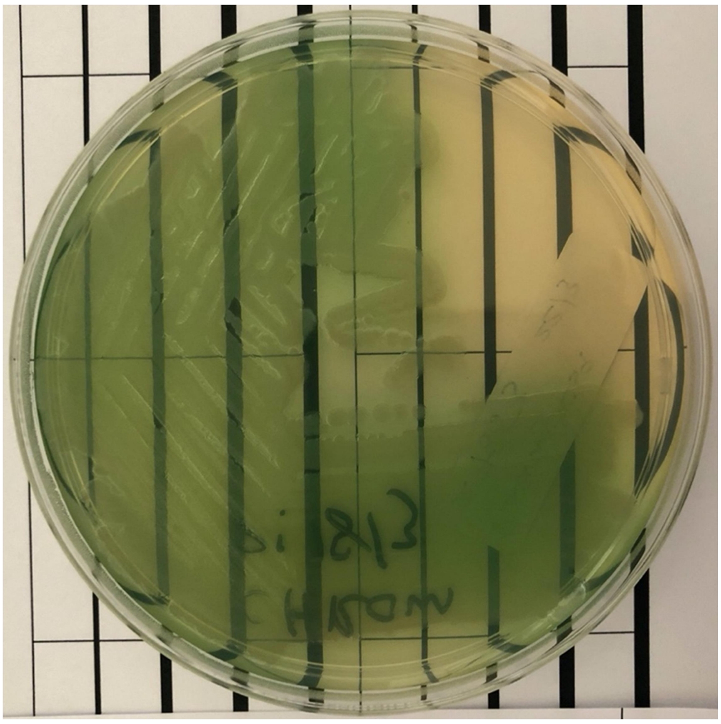

Figure 14. *Acinetobacter baumannii* ATCC 19606

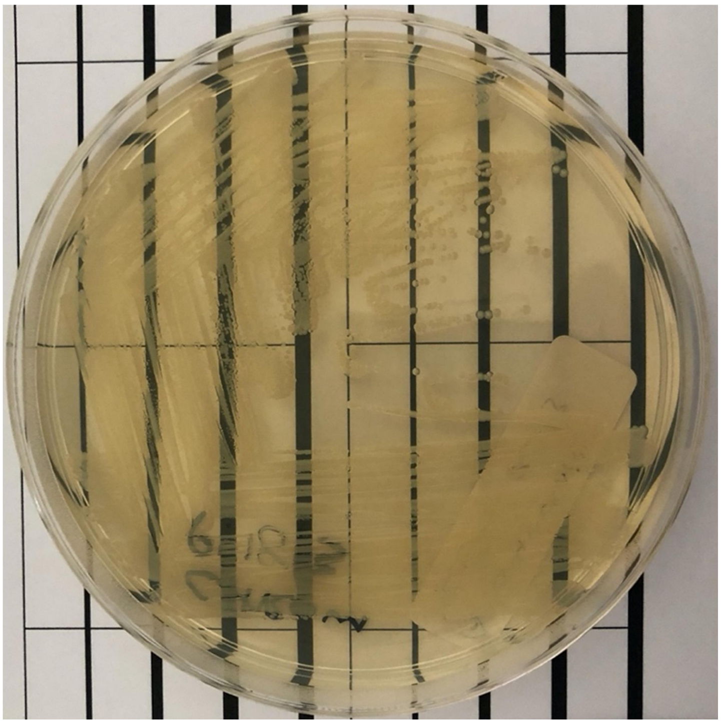

Figure 15. *Salmonella enterica* Enteritidis ATCC 13076

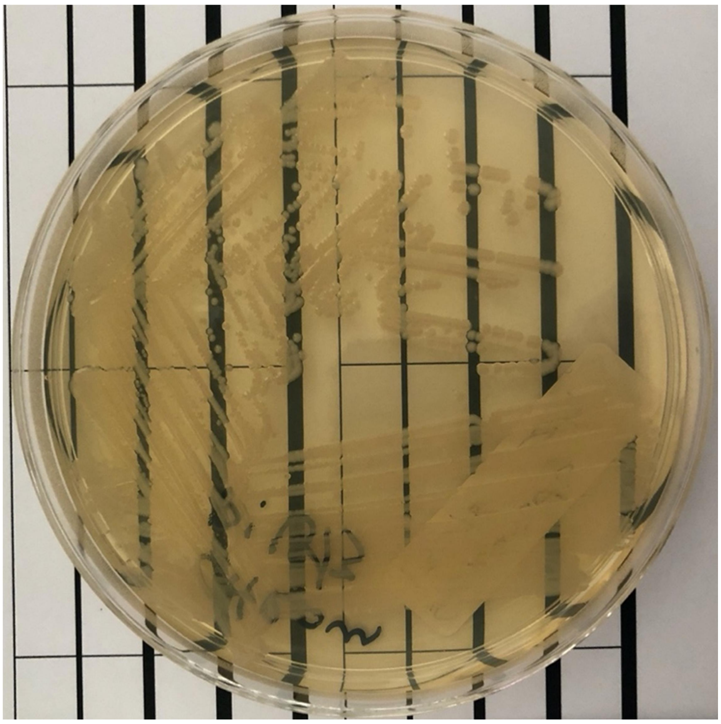

Figure 16. *Salmonella enterica* Typhimurium ATCC 14028

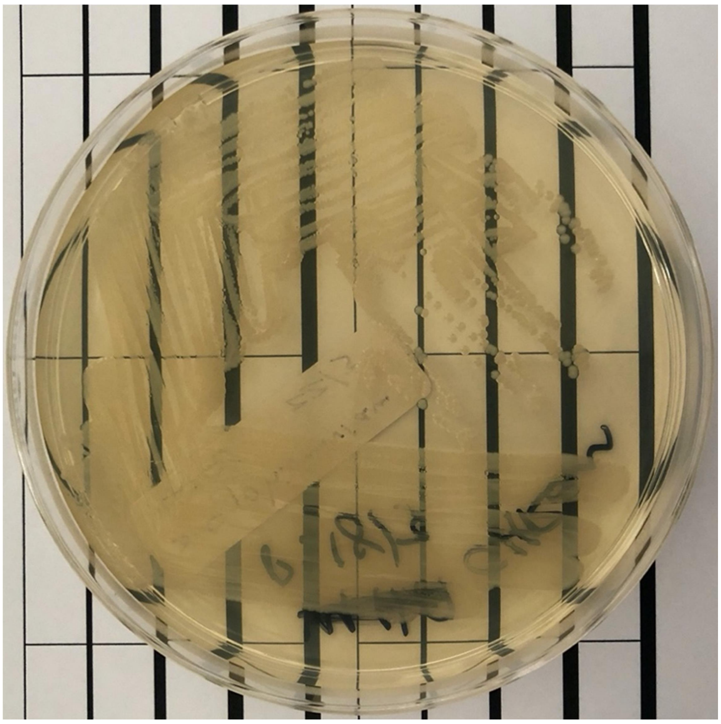

Figure 17. *Yersinia enterocolitica* subsp. *enterocolitica* ATCC 9610 (24 h)

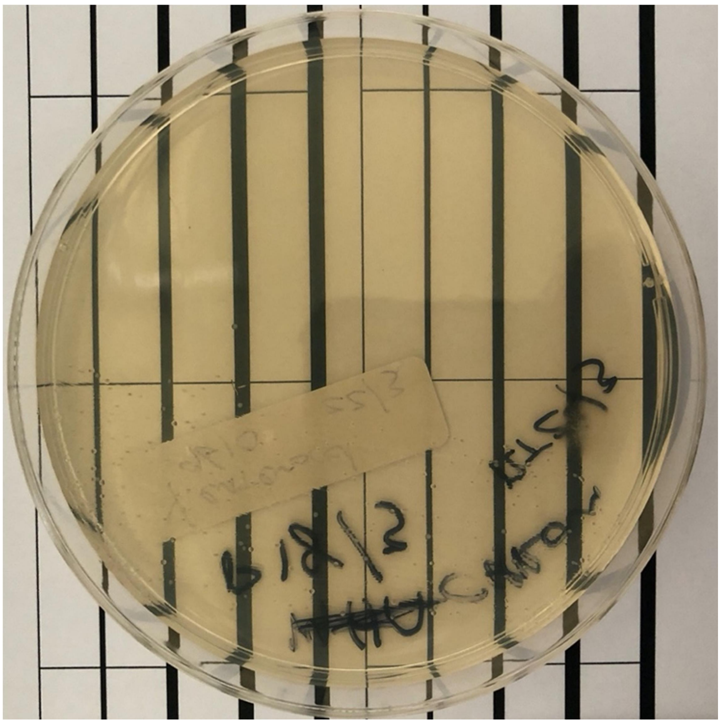

Figure 18. *Yersinia enterocolitica* subsp. *enterocolitica* ATCC 9610 (24 h at 37°C, followed by 24 h at room temperature)

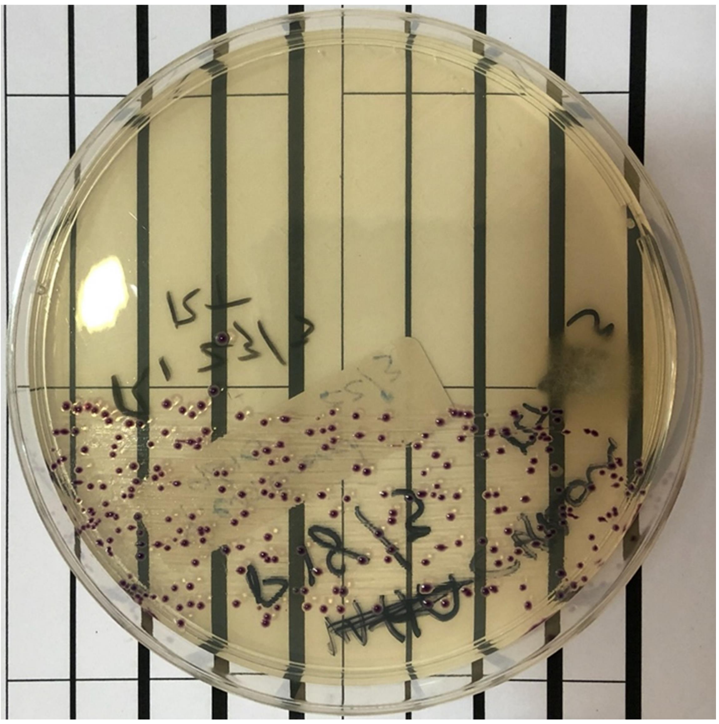

Figure 19. *Yersinia enterocolitica* subsp. *enterocolitica* ATCC 9610 (72 h)

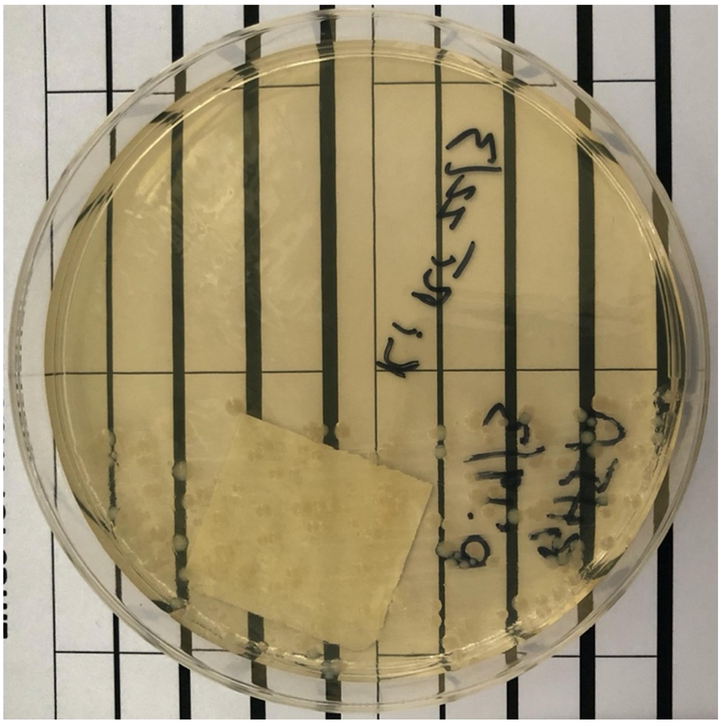

Figure 20. *Pseudomonas fluorescens* ATCC 13525 (24 h at 37°C followed by 48 h at room temperature)

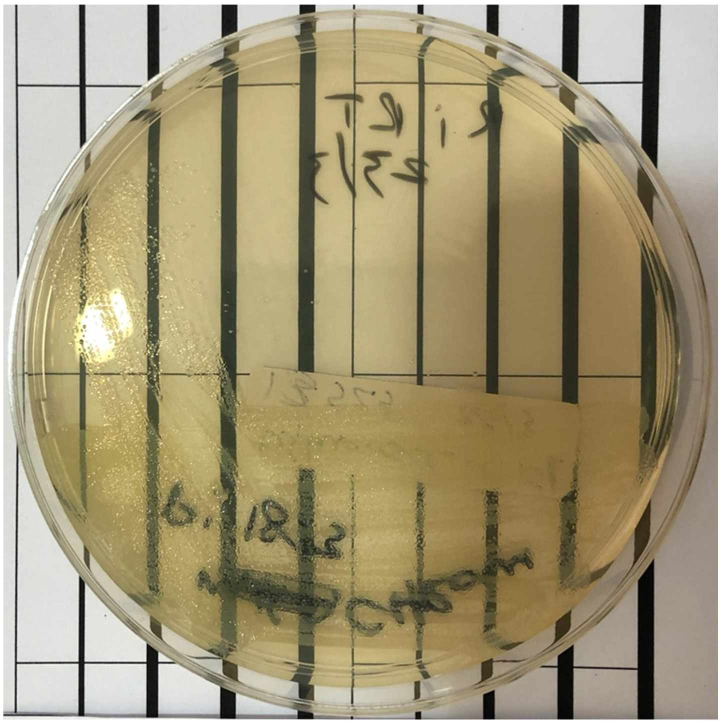

Figure 21. *Proteus mirabilis* ATCC 12453

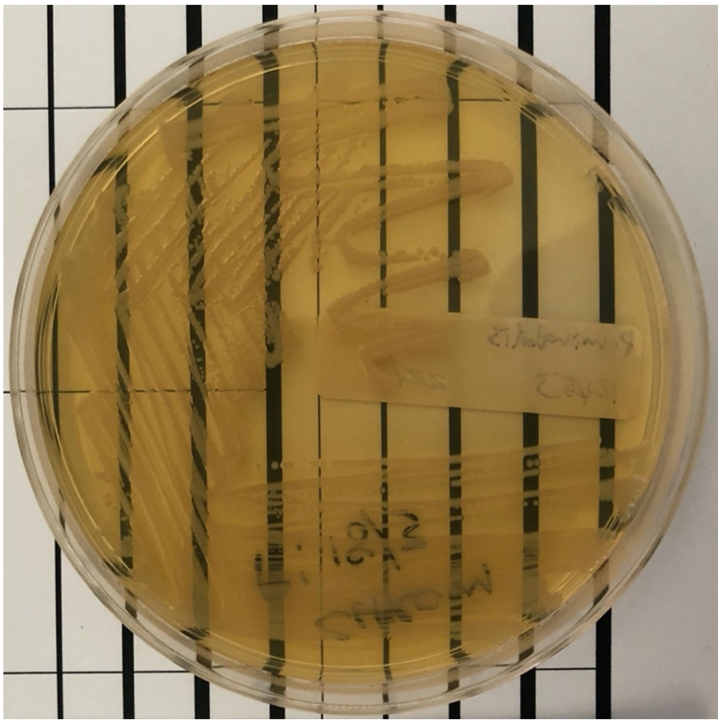

Figure 22. *Candida albicans* ATCC 10231

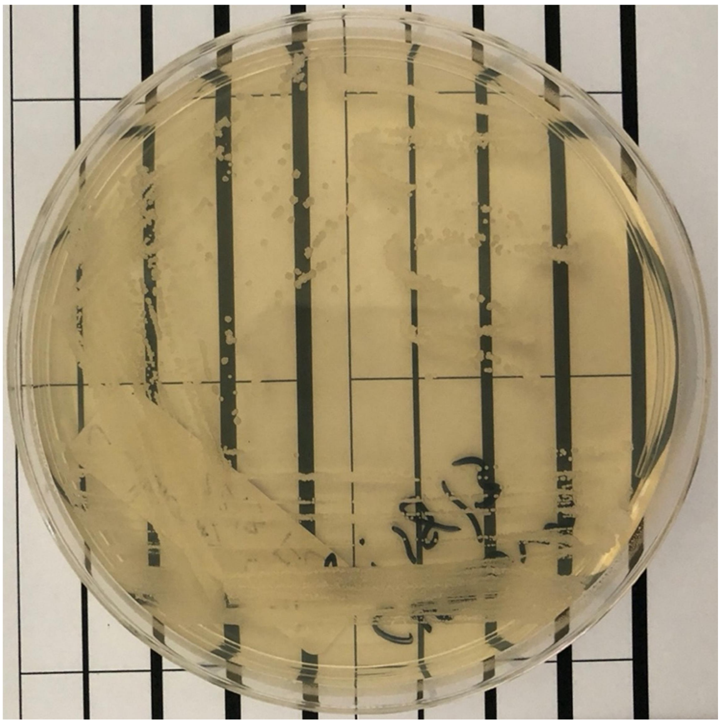

Figure 23. *Candida krusei* ATCC 34135

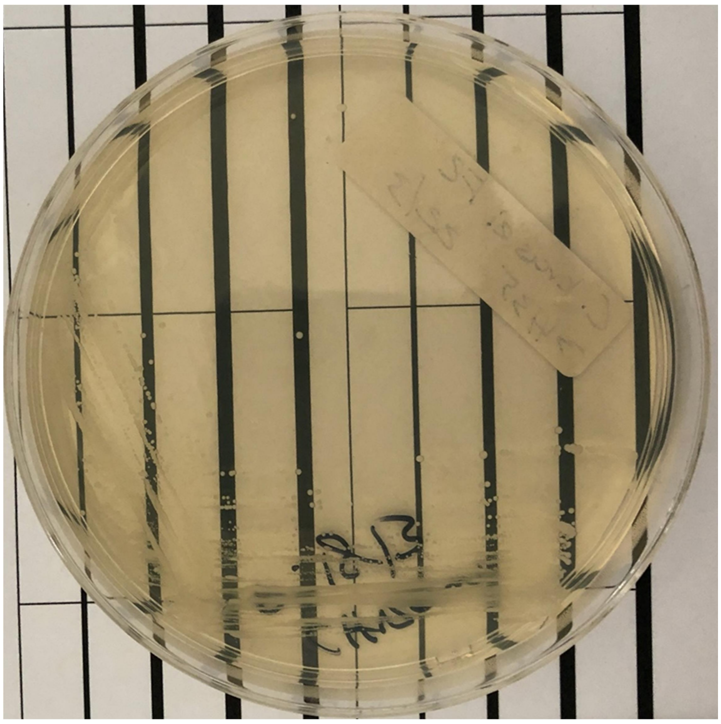

Figure 24. *Cryptococcus neoformans* ATCC 32045 (72 h)

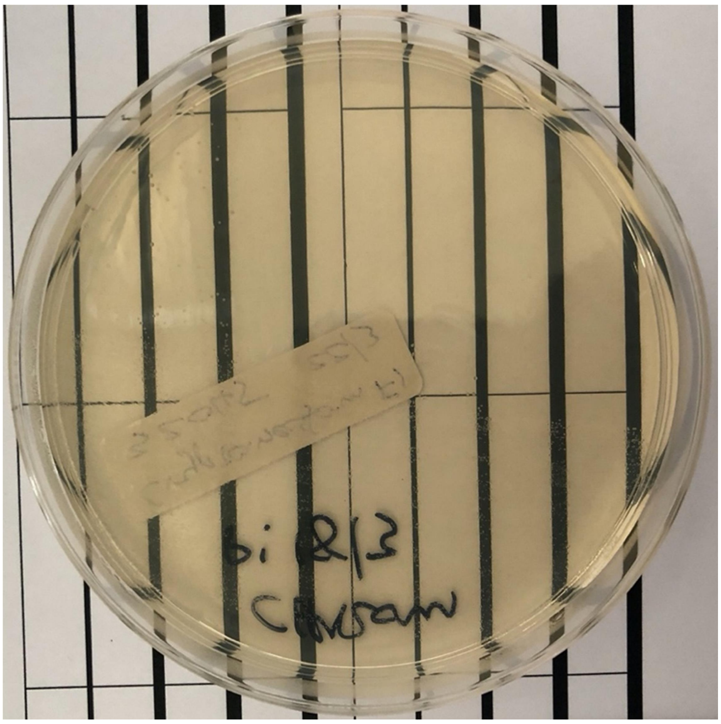

Supplementary Table 1. Comparison of direct disk diffusion on Chromatic MH agar to standard DD of laboratory isolates.

	K. pneumoniae				*K. pneumoniae*				*K. pneumoniae*			
	dDD		DD		dDD		DD		dDD		DD	
	mm	Int	mm	Int	mm	Int	mm	Int	mm	Int	mm	Int
AMC	11	**R**	14	**I**	13	**R**	15	**I**	15	**I**	18	**S**
AMP	6	R	6	R	6	R	6	R	6	R	6	R
CZ	6	R	6	R	6	R	6	R	6	R	6	R
FOX	15	**I**	24	**S**	16	**I**	21	**S**	14	**R**	21	**S**
CPD	6	R	6	R	ND				6	R	6	R
CAZ	11	R	16	R	15	R	18	**I**	15	**R**	19	**I**
CRO	6	R	11	R	6	R	6	R	6	R	6	R
CXM	6	R	6	R	6	R	6	R	6	R	6	R
C	17	**I**	27	**S**	16	I	17	I	14	**I**	20	**S**
CIP	25	**I**	29	**S**	6	R	6	R	6	R	6	R
GM	6	R	6	R	18	S	16	S	20	S	22	S
IMP	19	**R**	28	**S**	ND				20	**I**	28	S
MEM	22	**I**	29	**S**	20	I	22	I	20	**I**	28	**S**
RA	6	NA	9	NA	ND				6	NA	6	NA
TE	9	R	12	R	ND				12	**I**	17	**S**
SXT	6	R	6	R	19	S	16	S	17	S	22	S
NIT	15	**I**	14	**R**	ND				15	I	16	I

	S. enterica				*K. aerogenes*				*V. parahaemolyticus*			
	dDD		DD		dDD		DD		dDD		DD	
	mm	Int	mm	Int	mm	Int	mm	Int	mm	Int	mm	Int
AMC	21	S	27	S	10	R	9	R	25	S	29	S
AMP	19	S	26	S	6	R	9	R	22	S	26	S
CZ	22	**I**	26	**S**	6	R	6	R	22	NA	23	NA
FOX	21	S	26	S	6	R	6	R	23	S	21	S
CPD	23	S	29	S	ND				25	NA	25	NA

CAZ	24	S	28	S	21	S	25	S	30	S	26	S
CRO	26	S	33	S	25	S	26	S	29	NA	30	NA
CXM	19	S	24	S	22	S	21	S	22	S	19	S
C	21	S	25	S	23	S	23	S	30	S	29	S
CIP	25	**I**	32	**S**	29	**S**	25	**I**	24	S	26	S
GM	22	S	25	S	20	S	16	S	23	S	19	S
IMP	19	**R**	30	**S**	ND				ND		29	NA
MEM	26	S	34	S	25	S	24	S	33	S	33	S
RA	6	NA	6	NA	ND	ND	ND	ND	18	S	18	S
TE	6	R	6	R	ND	ND	ND	ND	25	S	25	S
SXT	15	S	21	S	25	S	23	S	28	S	28	S
NIT	14	R	13	R	ND	ND	ND	ND	ND		22	NA

	P. aeruginosa				*P. aeruginosa*				*A. baumannii*			
	dDD		DD		dDD		DD		dDD		DD	
	mm	Int	mm	Int	mm	Int	mm	Int	mm	Int	mm	Int
CAZ	20	S	25	S	6	R	6	R	20	S	19	S
CRO	19	NA	21	NA	6	NA	6	NA	15	**I**	13	**R**
CIP	25	**S**	24	**I**	6	R	6	R	23	S	25	S
GM	20	S	25	S	6	R	6	R	12	R	11	R
IMP	ND				6	R	6	R	ND			
MEM	24	S	30	S	6	R	6	R	26	S	26	S
SXT	ND				ND				6	R	6	R

	S. aureus			
	dDD		DD	
	mm	Int	mm	Int
AMC	15	NA	16	NA
AMP	6	NA	11	NA
AZM	6	R	6	R
CZ	14	NA	16	NA
FOX	11	R	11	R

CPD	6	NA	6	NA
CAZ	9	NA	10	NA
CRO	9	NA	10	NA
CXM	10	NA	14	NA
C	17	**I**	23	**S**
CIP	9	R	6	R
CC	21	S	29	S
E	6	R	9	R
GM	21	S	23	S
MEM	18	NA	19	NA
P	6	NA	9	NA
RA	26	S	35	S
TE	25	S	26	S
SXT	24	S	30	S
VA	19	NA	28	NA
NIT	23	S	24	S

dDD: Direct from positive blood bottle disk diffusion on Chromatic MH agar

DD: Standard disk diffusion testing

Int: Interpretation based on CLSI breakpoints.

S: Susceptible, I: Intermediate, R: Resistant

ND: Not Done

NA: No applicable CLSI breakpoint

Supplementary Table 2. Reproducibility of DD on Chromatic™ MH agar

	Expected		Measured (mm)				
	Range (mm)	Inter-pretation	Mean (mm)	SD (%CV) (mm)	Range (mm)	Inter-pretation	95% CI (mm)
***S. aureus* ATCC 25923 (*n*=4)**							
AMC	28-36	-[1]	34.3	1.3 (3.7)	33-36	-	32-36
AMP	27-35	-	35.3	0.5 (1.4)	35-36	-	34-36
AZM	**21-26**	S	24.0	2.4 (10.2)	21-26	S	**20-28**
CZ	29-35	-	31.3	1.3 (4)	30-33	-	29-33
FOX	23-29	S	25.3	1.0 (3.8)	24-26	S	24-27
CPD	19-25	-	23.3	0.5 (2.2)	23-24	-	22-24
CAZ	16-20	-	16.3	0.5 (3.1)	16-17	-	15-17
CRO	22-28	-	23.8	1.0 (4)	23-25	-	22-25
CXM	27-35	-	33.0	1.2 (3.5)	32-34	-	31-35
C	**19-26**	S	23.0	2.4 (10.6)	21-26	S	**19-27**
CIP	22-30	S	23.8	1.0 (4)	23-25	S	22-25
CC	24-30	S	25.7	0.6 (2.2)	25-26	S	25-27
E	22-30	S/I[2]	25.8	0.5 (1.9)	25-26	S	25-27
GM	**19-27**	S	21.8	2.2 (10.2)	20-25	S	**18-25**
MEM	29-37	-	32.5	1.3 (4)	31-34	-	30-35
P	26-37	-	35.5	0.6 (1.6)	35-36	-	26-35
RA	26-34	S	30.0	1.4 (4.7)	28-31	S	28-32
TE	24-30	S	25.0	0.8 (3.3)	24-26	S	24-26
SXT	**24-32**	S	26.5	1.9 (7.2)	24-28	S	**23-30**
VA	**17-21**	-	16.0	0.8 (5.1)	**15-17**	-	**15-17**

NIT	18-22	S	19.0	0.8 (4.3)	18-20	S	18-20
***E. coli* ATCC 25922 (*n*=8)**							
**AMC**	**18-24**	S	17.9	1.1 (6.3)	**16-19**	Sx5, **Ix3**	**17-19**
AMP	15-22	S/I[3]	17.0	1.4 (8.3)	15-19	Sx4, Ix4	16-18
**CZ**	**21-27**	S/I[4]	20.4	0.9 (4.5)	**19-22**	Ix7, **Rx1**	**20-21**
FOX	23-29	S	23.9	1.0 (4.2)	23-26	S	23-25
CPD	**23-28**	S	23.2	1.7 (7.4)	**20-25**	S	**22-25**
CAZ	25-32	S	27.1	1.6 (6.1)	25-29	S	26-28
CRO	**29-35**	S	29.6	3.1 (10.5)	**25-34**	S	**27-32**
CXM	**20-26**	S	21.0	1.5 (7.2)	**18-23**	S	20-22
**C**	**21-27**	**S**	24.1	2.1 (8.7)	**20-26**	Sx6, **Ix2**	22-26
CIP	**30-40**	S	28.6	3.3 (11.5)	**24-32**	S	**26-31**
GM	**19-26**	S	20.4	2.4 (11.7)	**16-23**	S	18-22
IMP	**26-32**	S	26.8	1.3 (5.0)	**25-28**	S	26-28
MEM	**28-35**	S	27.9	2.6 (9.5)	**25-33**	S	**26-30**
RA	**8-10**	-	9.5	0.8 (8.8)	**9-11**	-	9-10
TE	**18-25**	S	22.7	3.2 (14.1)	**20-29**	S	20-25
SXT	23-29	S	22.1	1.4 (6.1)	**20-23**	S	**21-23**
NIT	**20-25**	S	19.0	1.3 (6.9)	**18-21**	S	**18-20**
***E. coli* ATCC 35218 (*n*=8)**							
AMC	17-22	S/I[5]	17.8	1.4 (7.8)	**16-20**	Sx5, Ix3	17-19

AMP	6	R	6.0	0.0 (0.0)	6	R	6
***P. aeruginosa* ATCC 27853 (*n*=10)**							
CAZ	**22-29**	S	25.8	2.7 (10.3)	**20-29**	S	24-28
CRO	**17-23**	-	19.4	3.2 (16.3)	**11-22**	-	17-22
CIP	25-33	S	28.3	2.0 (7.1)	25-31	S	27-30
GM	**19-23**	S	19.2	0.8 (4.1)	**18-20**	S	19-20
IMP	20-28	S	22.1	1.1 (5.1)	21-24	S	21-23
MEM	**27-33**	S	28.2	1.7 (6.0)	**24-30**	S	27-29

[1]No CLSI breakpoints for disk diffusion testing

[2]CLSI breakpoints: S ≥ 23, I 14-22, R ≤ 13

[3]CLSI breakpoints: S ≥ 17, I 14-16, R ≤ 13

[4]CLSI breakpoints for infections other than uncomplicated UTIs: S ≥ 23, I 20-22, R ≤ 19

[5]CLSI breakpoints S ≥ 18, I 14-17, R ≤ 13
